# Daily vancomycin dose requirements as a continuous infusion in obese versus non-obese SICU patients

**DOI:** 10.1186/s13054-016-1363-9

**Published:** 2016-07-01

**Authors:** Hsin Lin, Daniel Dante Yeh, Alexander R. Levine

**Affiliations:** Department of Pharmacy, Massachusetts General Hospital, 55 Fruit Street, Boston, MA 02114 USA; Departments of Surgery, Massachusetts General Hospital, 55 Fruit Street, Boston, MA 02114 USA; Department of Pharmacy Practice and Administration, University of Saint Joseph School of Pharmacy, 229 Trumbull St, Hartford, CT USA

**Keywords:** Vancomycin continuous infusion, Obesity, Pharmacokinetic and pharmacodynamic, Infectious disease

## Abstract

**Background:**

Limited data are available assessing vancomycin concentrations in obese critically ill patients. Currently, there are no studies evaluating dosing requirements in this population who receive vancomycin administered as a continuous infusion (CI). The aim of this study was to assess whether there was a difference in the weight-based maintenance dose required to reach a therapeutic vancomycin concentration at 24 hours when given as a CI in obese versus non-obese critically ill patients.

**Methods:**

A retrospective cohort study of adult obese patients admitted to the SICU between 2013 and 2015 receiving a vancomycin CI (CIV), and with 24-hour serum measurements were included. Obese patients (body mass index (BMI) ≥35 kg/m^2^) were matched with non-obese patients (BMI <30 kg/m^2^) based on renal function, age and acute physiology and chronic health evaluation (APACHE)-II score at admission. All patients in this study received a loading dose of 25 mg/kg then a maintenance dose based on renal function according to the protocol. The study was approved by the Institutional Review Board. The primary outcome was the weight-based total daily maintenance dose required to achieve a vancomycin level of 20 mg/L. The secondary endpoints included the achievement of a therapeutic level at 24 hours.

**Results:**

Twenty-six matched pairs of patients met the inclusion criteria. Of these, 17 pairs had preserved renal function and 9 pairs required continuous venovenous hemofiltration. Mean BMI was 40.9 kg/m^2^ in obese and 24.8 kg/m^2^ in non-obese patients. To achieve a vancomycin concentration of 20 mg/L, the weight-based daily maintenance dose in obese patients was 25.6 mg/kg versus 43.8 mg/kg in non-obese patients (*p* <0.01). Therapeutic 24-hour levels were achieved in 24/26 obese versus 23/26 no-obese patients (*p* = 0.63). Mean 24-hour vancomycin level was 20.3 ± 3.81 mcg/ml in obese compared to 20.03 ± 3.79 mcg/ml in non-obese patients (*p* = 0.77). Mean daily maintenance doses required to achieve a level of 20 mcg/ml were 2961 ± 1670 mg in obese compared to 3189 ± 1600.69 mg in non-obese (*p* = 0.61).

**Conclusions:**

The results of our study suggest that critically ill obese patients treated with CIV required a significantly lower maintenance dose per unit of body weight than non-obese patients to achieve the same target level.

## Background

The prevalence of obesity has more than doubled worldwide since 1980 and it is estimated that 60 % of the world’s population will be classified as overweight or obese by the year 2030 [1]. Given this trend, it is important to characterize the effect of obesity on drug disposition. Critically ill patients are at risk of alterations to pharmacokinetics due to having a larger volume of distribution and alteration in protein binding [2]. Several physiologic changes in obesity affect antimicrobial pharmacokinetics. For example, the volume of distribution in obese patients is greater due to increased lean body mass and adipose tissue [3]. Drug clearance may also be further enhanced due to increased kidney mass and filtration [4].

Optimizing antibiotic dosing is a priority in critically ill patients because inadequate systemic concentrations may lead to treatment failure and development of antimicrobial resistance [5]. It is imperative that the initial dosing regimens provide adequate pharmacodynamic exposure for the most likely pathogen. Vancomycin is the most common first-line option for treating methicillin-resistant *Staphylococcus aureus* or other resistant Gram-positive bacteria, such as coagulase-negative staphylococci and ampicillin-resistant enterococci in severe healthcare-associated infections.

Despite an increasing number of obese patients worldwide, there is little information on how to optimally dose vancomycin in these patients. Few studies have evaluated vancomycin concentrations in obese critically ill patients, and studies are limited by small patient groups and utilization of standard intermittent vancomycin dosing [4, 6–10]. Currently, there are no studies evaluating dosing requirements in this population when receiving vancomycin continuous infusions (CIV). The aim of this study was to determine the weight-based dosing requirement of CIV necessary to reach the 24-hour target concentration in obese versus non-obese critically ill patients.

## Methods

We reviewed all adult patients (≥18 years old) who received CIV, either as monotherapy or combined with other antibiotics, in our multidisciplinary surgical ICU between January 2013 and January 2015. Patients were identified using the pharmacy informatics system. We included all adult patients who had a body mass index (BMI) ≥35 kg/m^2^, and had measurement of serum vancomycin concentrations 24 hours after treatment. Patients who had previously received vancomycin within 48 hours prior to the start of the continuous infusion (CI) were excluded. The protocol was approved by the Partners Institutional Review Board, which waived the need for informed consent because of the retrospective nature of the study.

Demographic data (age, sex, total body weight (TBW), ideal body weight (IBW), adjusted body weight (ABW), height, BMI, creatinine clearance upon vancomycin initiation (CrCL), severity of illness score (acute physiology and chronic health evaluation (APACHE) II) calculated at the time of ICU admission, and laboratory parameters were retrospectively collected for each patient in the study. ABW was calculated as follows:

$$ \mathrm{A}\mathrm{B}\mathrm{W} = 0.4\ \left(\mathrm{T}\mathrm{B}\mathrm{W}\ \hbox{--}\ \mathrm{I}\mathrm{B}\mathrm{W}\right) + \mathrm{I}\mathrm{B}\mathrm{W}. $$

TBW was estimated and measured by the nurse using the patient’s bed scale on the day of vancomycin therapy. Other gathered data included use of continuous venovenous hemofiltration (CVVH), CVVH hemofiltration rate, use of vasopressors or mechanical ventilation, vancomycin dose, and serum vancomycin concentration. Nephrotoxicity was defined as an increase in serum creatinine (SCr) by 0.5 mg/dL or at least a 50 % increase from baseline over two consecutive SCr values for patients not requiring CVVH.

CI vancomycin clearance (CLvanc) was calculated using the following equation:

$$ \frac{\mathrm{Dose}\ \left(\mathrm{mg}/\mathrm{hr}\right)}{\mathrm{Serum}\ \mathrm{concentration}\ \left(\mathrm{mg}/\mathrm{L}\right)}=\mathrm{CLvanc}\ \left(\mathrm{L}/\mathrm{hr}\right) $$

Area under the concentration curve 24 hr (AUC24) was calculated as follows:

$$ \mathrm{serum}\ \mathrm{vancomycin}\ \mathrm{concentration}\ \left(\frac{\mathrm{mg}}{\mathrm{L}}\right)*24\mathrm{hr} $$

Daily vancomycin dose (mg) needed to obtain a target concentration of 20 mg/L while on CI:

$$ \mathrm{target}\ \mathrm{concentration}\kern0.5em 20\ \left(\frac{\mathrm{mg}}{\mathrm{L}}\right)*\mathrm{CLvanc}\ \left(\frac{\mathrm{L}}{\mathrm{hr}}\right)*24\mathrm{hr} $$

The 24-hour urine creatinine clearance (CrCL) was calculated using the following formula:

$$ \mathrm{CrCL}\ \left(\frac{\mathrm{ml}}{ \min}\right)=\frac{\left(\mathrm{Urine}\ \mathrm{output},\ \mathrm{ml}\right)*\left(\mathrm{Urinary}\ \mathrm{creatinine},\frac{\mathrm{mg}}{\mathrm{dL}}\right)}{\left(\mathrm{serum}\ \mathrm{creatinine},\frac{\mathrm{mg}}{\mathrm{dL}}\right)*\left(\mathrm{Time}\ \mathrm{of}\ \mathrm{urine}\ \mathrm{collection},\ \mathrm{minutes}\right)} $$

All CVVH treatments applied the CAR-505 filter with a polyethersulfone membrane with a 1.6 m^2^ membrane surface area in conjunction with the NxStage System One dialysis machine. Vancomycin concentration analysis utilized a Syva Emit 2000 vancomycin assay (Siemens Healthcare Diagnostics, Inc. Newark, DE, USA). The assay has an analytical range between 5.0 and 50.0 mcg/ml, and the between-run coefficient of variation was <10 % throughout the analytical range.

### Vancomycin treatment and measurements

In our surgical ICU, the use of CIV is at the discretion of the treating ICU physician and at a loading dose of 25–30 mg/kg, followed by maintenance doses adjusted based on CrCL or CVVH. Calculated CrCL was estimated using the Cockcroft-Gault (CG) equation using ABW. To account for a falsely elevated CrCL estimate in patients >65 years of age in whom low SCr values may indicate reduced muscle mass, SCr values <0.8 mg/dL were adjusted up to 0.8 mg/dL. CG CrCL was used in the dosing nomogram because 24-hour urine creatinine cannot provide immediate results and rapid administration of antibiotic is often essential. Of note, 24-hour urine creatinine is not performed daily in the study center, instead it is collected on the first day of CIV therapy. The daily maintenance dose starts at 2000 mg for CG CrCL of 60 ml/minute; increases in CG CrCL of 10 ml/minute requires an increase in maintenance dose of 250 mg vancomycin. The maximum maintenance dose is started at 4250 mg. For patients who receive concomitant CVVH, the initial vancomycin dose is 1500 mg daily.

Doses were not changed during the first 24 hours of therapy; afterwards, the daily regimen was adapted using a specific approach: if serum vancomycin concentration was <15 mg/L, an additional dose of 500 to 1000 mg was given as a bolus followed by an increased total daily dose proportional to the goal serum concentration. If the concentration was >25 mg/L, the CIV was discontinued for 4 to 6 hours and resumed with the daily dose reduced proportionally. Serum vancomycin concentrations were determined every 24 hours until two serum concentrations were within target range (15–25 mg/L). If CVVH was stopped due to a clotted circuit, the CIV was stopped and the infusion resumes once CVVH was restarted.

### Matched controls

Using an institutional database of all ICU patients who received CIV during the same period, obese ICU patients (BMI >35 kg/m^2^) were matched with non-obese ICU patients (BMI <30 kg/m^2^) according to three criteria: (1) renal function (either the same 24-hour urine CrCL with a range of eligibility for matching of 25 ml/minute, or if on CVVH, the same CVVH intensity with a range of eligibility for matching of 10 ml/kg/minute); (2) age (range of eligibility for matching of 15 years); and (3) APACHE II score at admission (range of eligibility for matching of 10).

### Endpoints

The primary outcome was the weight-based daily maintenance dose requirement to achieve vancomycin concentration of 20 mg/L at 24 hours. Secondary outcomes were achievement of a therapeutic level by 24 hours, CLvanc/IBW, CLvanc/ABW, CLvanc/TBW, and AUC24. Therapeutic levels were defined as a range of 15 to 25 mg/L to achieve an adequate AUC [11, 12].

### Statistical analysis

Distributions of quantitative outcomes were summarized by the mean with standard deviation and were compared using the Mann-Whitney *U* test. Categorical outcomes were summarized by counts and proportions and compared using the Chi-square test (or the Fisher’s exact test as appropriate). Linear regression was used to identify the correlation between CLvanc and CrCL. All analyses were performed using STATA Data Analysis and Statistical Software (version 13; StatCorp LP, College Station, TX, USA). A *p* value <0.05 was considered statistically significant.

## Results

Twenty-six matched pairs of patients met the inclusion criteria. Of these, 17 pairs of patients had preserved renal function and 9 pairs required CVVH. Baseline values of ABW, TBW, and percent over IBW and BMI were significantly different (Table 1). Mean BMI was 41 kg/m^2^ in obese and 24.9 kg/m^2^ in non-obese patients. In patients with preserved renal function (*n* = 34), mean 24-hour urine CrCL was 166 ml/minute in obese patients versus 168 ml/minute in non-obese patients. In patients (*n* = 18) receiving CVVH, mean hemofiltration rates were 21.3 ml/kg/h in obese patients versus 21.9 ml/kg/h in non-obese patients. No patients in either group with preserved renal function developed nephrotoxicity during vancomycin treatment.

**Table 1:** Demographic data

	Obese patients (*n* = 26)	Non obese patients (*n* = 26)	*P* value
Age, years	54.2 (16.8)	55.4 (16.0)	0.781
Male, *n*	18	21	0.337
TBW, kg	117 (23.3)	75.2 (13.3)	<0.001
ABW, kg	87.7 (13.7)	71.0 (11.1)	<0.001
IBW, kg	67.9 (13.1)	68.5 (10.5)	0.88
Height, in	67.6 (4.63)	68.5 (3.38)	0.393
Over IBW, %	1.77 (0.43)	1.1 (0.112)	<0.001
BMI, kg/m^2^	41.0 (8.12)	24.9 (3.16)	<0.001
APACHE II	20.3 (9.24)	18.7 (8.46)	0.524
Hospital mortality, *n*	5	4	0.714
Mechanical ventilation, *n*	24	26	0.149
Use of vasopressors, *n*	15	20	0.139
CVVH, *n*	9	9	1
Urine creatinine clearance (ml/min), *n* = 17	166 (62.7)	168.1 (59.1)	0.92
CVVH flow rate, ml/kg/h	21.3 (7.09)	21.9 (6.47)	0.86
Duration of vancomycin, days	6.88 (3.02)	6.5 (3.06)	0.415
Time from ICU admission to vancomycin initiation, days	2.73 (1.66)	2.53 (1.33)	0.647
Infection site - Pneumonia - Bacteremia - SSTI - Intraabdominal - Unknown	16 2 2 3 3	19 0 3 2 2	0.375 0.149 0.638 0.638 0.638
Microbiology results - MRSA - MSSA - Enterococcus - Streptococcus spp. - CoN Staphylococcus - Other	6 2 0 2 4 12	7 3 1 3 3 9	0.749 0.638 0.313 0.638 0.685 0.397

Results presented as mean (SD) unless stated otherwise. *CVVH* continuous venovenous hemofiltration, *TBW* total body weight, *ABW* adjust body weight, *IBW* ideal body weight, *BMI* body mass index, *APACHE* Acute Physiology and Chronic Health Evaluation, *SSTI* skin soft tissue infection, *MRSA* methicillin-resistant *Staphylococcus aureus*, MSSA, methicillin-sensitive *Staphylococcus aureus*

### Endpoints

For the primary endpoint, the weight-based daily maintenance dose required to achieve a therapeutic 24-hour serum concentration in obese patients was 24.8 mg/kg versus 41.9 mg/kg in non-obese patients (*p* < 0.001) (Table 2). There was no difference between groups in the mean 24-hour vancomycin serum concentration, mean daily maintenance doses, AUC24, or achievement of therapeutic concentration within 24 hours. Mean loading doses were 24.8 mg/kg versus 25.6 mg/kg in obese and non-obese patients, respectively, *p* = 0.135.

**Table 2:** Vancomycin dose and treatment

	Obese (*n* = 26)	Non obese (*n* = 26)	*P* value
Loading dose, mg	2894 (525)	1923 (345)	<0.001
Loading dose, mg/kg	24.8 (2.05)	25.6 (1.66)	0.135
Maintenance dose, mg	2885 (1450)	3039 (1313)	0.69
Maintenance dose, mg/kg	24.8 (12.7)	41.9 (20)	<0.001
24 hour serum concentration, mg/L	20.3 (3.81)	20.0 (3.79)	0.777
CLvanc, L/h	6.16 (3.47)	6.64 (3.33)	0.617
Dose needed to achieve concentration of 20 mg/L, mg	2961 (1670)	3189 (1601)	0.617
Dose per ABW needed to achieve concentration of 20 mg/L, mg/kg	25.57 (14.8)	43.8 (23.5)	<0.01
AUC24	488. (91.5)	480.9 (91.04)	0.777
Achievement of therapeutic concentration at 24 hours, *n*	24	23	0.638

Results are presented as mean (SD) or number. *CLvanc* continuous infusion vancomycin clearance, *ABW* adjusted body weight, *AUC24* area under the concentration curve 24 h

In the subgroup of 18 patients who received CVVH, there was no difference in the CLvanc/ABW or CLvanc/IBW (Table 3). CLvanc/TBW was significantly lower in obese than non-obese patients, *p* = 0.04. There was no correlation between CLvanc and TBW, IBW or ABW. There was strong positive correlation between CLvanc (L/h) and CVVH flow rate in obese patients (*r*^2^ = 0.93, *p* < 0.001) but poor correlation in non-obese patients (*r*^2^ = 0.2, *p* = 0.226).

**Table 3:** Vancomycin dose and monitoring during CVVH

	Obese patients (*n* = 9)	Non obese patients (*n* = 9)	*P* value
Goal concentration, 24 h, *n*	9	9	1
Serum concentration, 24 h, mg/L	19.8 (3.05)	21.6 (3.02)	0.155
Dose needed to achieve concentration of 20 mg/L, mg	1426 (418)	1383 (282)	0.802
Dose needed to achieve concentration of 20 mg/L, mg/kg	13.8 (5.09)	19.9 (6.7)	0.004
Loading dose, mg	2722 (441)	1833 (280)	<0.001
Loading dose, mg/kg	25.3 (0.774)	25.4 (1.49)	0.847
Maintenance dose, mg	1389 (397)	1500 (375)	0.550
Maintenance dose, mg/kg	13.3 (4.73)	21.7 (8.33)	0.01
AUC24	474 (73.2)	517 (48.6)	0.15
CLvanc, L/h	2.97 (0.87)	2.88 (0.60)	0.802
CLvanc/ABW, L/h/kg	0.04 (0.01)	0.04 (0.01)	0.34
CLvanc/IBW, L/h/kg	0.02 (0.02)	0.05 (0.01)	0.879
CLvanc/TBW, L/h/kg	0.03 (0.01)	0.04 (0.01)	0.044

Results are presented as mean (SD) unless stated otherwise. *CVVH* continuous venovenous hemofiltration, *AUC24* area under the concentration curve 24 h, *CLvanc* continuous infusion vancomycin cl, *ABW* adjusted body weight, *IBW* ideal body weight, *TBW* total body weight

**Fig. 1:**
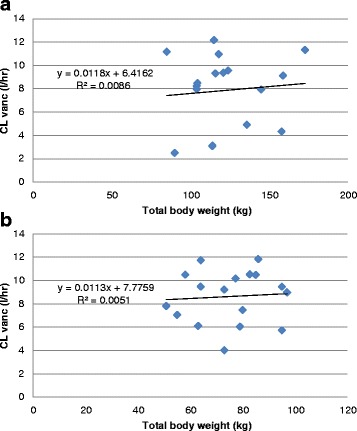
**a** Correlation of continuous infusion vancomycin clearance (*CLvanc*) and total body weight in obese patients (*n* = 26). **b** Correlation of CLvanc and total body weight in non-obese patients (*n* = 26)

**Fig. 2:**
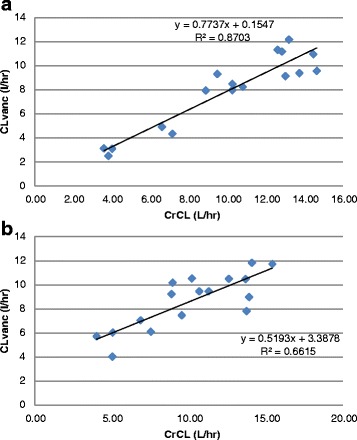
**a** Correlation between continuous infusion vancomycin clearance (*CLvanc*) and creatinine clearance (*CrCL*) in obese patients with preserved renal function (*n* = 17). **b** Correlation between CLvanc and CrCL in non-obese patients with preserved renal function (*n* = 17)

In the subgroup of 34 patients with preserved renal function, obese patients had significantly lower CLvanc/CrCL than non-obese patients, *p* = 0.04 (Table 4). Mean CLvanc in the obese group was 0.06 L/h per kg TBW, whereas non-obese patients had a CL of 0.12 L/h per kg TBW. There were no significant differences between obese and non-obese patients in CLvanc and CLvanc adjusted for IBW. There was no correlation between CLvanc and TBW in any patients as shown in Fig. 1a and b. There was good correlation between vancomycin CL and CrCL in obese patients (*r*^2^ = 0.87 *p* < 0.001) and non-obese patients (*r*^2^ = 0.661, *p* < 0.001) as shown in Fig. 2a and b.

**Table 4:** Vancomycin dosing and monitoring in patients with preserved renal function

	Obese patients (*n* = 17)	Non-obese patients (*n* = 17)	*P* value
Goal concentration, 24 h, *n*	15	14	0.628
Serum concentration, 24 h, mg/L	20.6 (4.21)	19.6 (4.41)	0.494
Dose needed to achieve concentration of 20 mg/L, mg	3774 (1498)	4141 (1087)	0.420
Dose needed to achieve concentration of 20 mg/L, mg/kg	31.8 (14.5)	56.9 (17.9)	<0.001
Loading dose, mg	2985 (555)	1941 (370)	<0.001
Loading dose, mg/kg	24.6 (2.47)	25.9 (1.88)	0.09
Maintenance dose, mg	3676 (1131)	3882 (674)	0.523
Maintenance dose, mg/kg	30.8 (11.3)	53.4 (13.7)	<0.001
AUC24	496 (101)	471 (106)	0.494
CLvanc, L/h	7.86 (3.11)	8.62 (2.26)	0.420
CLvanc/CrCL	0.79 (0.11)	0.91 (0.21)	0.049
CLvanc/ABW, L/h/kg	0.09 (0.04)	0.12 (0.04)	0.0074
CLvanc/IBW, L/h/kg	0.12 (0.05)	0.12 (0.04)	0.404
CLvanc/TBW, L/h/kg	0.06 (0.03)	0.12 (0.04)	0.0001

Results are presented as mean (SD) unless stated otherwise. *AUC24* area under the concentration curve 24 h, *CLvanc* continuous infusion vancomycin clearance, *CrCL* creatinine clearance, *ABW* adjusted body weight, *IBW* ideal body weight, *TBW* total body weight

## Discussion

To our knowledge, this is the first study to evaluate the dosing requirements of CIV in obese critically ill patients compared to a non-obese cohort. Our study has several key findings. First, the daily weight-based dose of CIV is significantly lower for obese patients than non-obese patients, thereby, reducing total drug exposure and potentially lowering their risk of kidney injury. Second, vancomycin clearance is similar in obese and non-obese patients and is poorly correlated with TBW. Finally, our results highlight that our CIV dosing protocol reliably achieved therapeutic concentrations in both obese and non-obese critically ill patients.

### Continuous vancomycin infusions reduce total daily drug exposure in obese patients

CIV has been proposed as an alternative approach to optimize drug concentrations and efficacy in various critically ill patient populations. Although CI has not been proven to be superior to intermittent infusion for clinical effectiveness, CI enhances the probability of achieving optimal drug exposure and lessens the renal toxicity potential associated with more aggressive dosing [13]. For example, Hanrahan et al. demonstrated that patients who received intermittent vancomycin infusions had 8.2 times greater odds of developing nephrotoxicity compared to CIV [14]. The risk of nephrotoxicity is further amplified in obese patients who traditionally receive significantly higher doses.

### Vancomycin clearance in obese patients is similar in non-obese patients and is not significantly associated with TBW

Previous studies have identified greater CLvanc in obese patients with normal serum creatinine [8, 9, 15]. As obese patients have a larger volume of distribution (Vd), they may have a higher CrCL and CLvanc. It has also been theorized that obese patients have a larger Vd due to greater quantity and size of nephrons and increased blood flow to the kidney [16, 17]. Bauer et al. demonstrated that the extent of CLvanc increased significantly more than the increase in Vd in morbidly obese patients, suggesting a much shorter half-life [8]. However, in our study, we found no difference in CLvanc between obese and non-obese patients with preserved renal function. After adjusting CLvanc for various body weights, we observed no difference in CLvanc/IBW. This may imply that the potential increase in Vd in obese patients does not change proportionally to CLvanc.

Consensus guidelines recommend using TBW to guide vancomycin dosing because data indicate that TBW predicts CLvanc in obese individuals [18]. Contrary to the previous findings we did not identify correlation between CLvanc and TBW in either group [7, 8]. In our study the only variables statistically significantly associated with CLvanc were urine CrCL and CVVH hemofiltration rate. Both obese and non-obese patients with the same urine CrCL or CVVH hemofiltration rate required the same total daily vancomycin as a CIV to achieve the target concentration. Differences in our study population are a plausible explanation for these results: in previous studies, patients had TBW at least 90 % over the IBW and had higher BMI compared to the patients in our study. The consequences of varying Vd in obesity can affect CLvanc and its relationship with TBW. Second, our study population represents critically ill patients who may have greater non-renal clearance compared to a non-ICU population. Therefore, when utilizing CIV in critically obese patients, our data suggest that clinicians should rely on 24-hour measured CrCl or the CVVH hemofiltration rate rather than TBW to assess CLvanc. Additional studies are needed to confirm these findings.

The strengths of our study include a well-matched cohort of obese and non-obese critically ill patients, use of a standardized CIV protocol, and incorporation of 24-hour measured CrCl to estimate renal clearance of vancomycin. Cockcroft-Gault CrCL (CG CrCL) is one of the most commonly used methods for estimating renal function in the ICU. Most pharmacokinetics studies in critically ill patients rely on CG CrCL to estimate drug clearance and adjust drug dosages. However, the use of CG CrCL to estimate CrCL raises several concerns as it has been repeatedly shown to be inaccurate in the critical care setting, in particular, among patients with augmented renal clearance [19]. Only measured urine CrCL should be used to accurately guide drug dosing [20].

Our study is limited by its retrospective nature, as the data are subject to inherent biases and small sample size. Our patient population was representative of younger, obese critically ill surgical patients with high illness severity scores and mortality rates. Although our obese cohort was well-matched with a non-obese cohort, the subgroup of patients with preserved renal function had high mean creatinine clearance. Therefore, our findings may not be applicable to older, non-surgical ICU patients with impaired renal function. Additionally, the precision of the measurement of TBW in the study patients cannot be confirmed. Many confounders, such as the presence of a sheet, pillow, blanket and equipment on the patient’s bed can falsely affect weight obtained from the bed scale and may not accurately reflect true TBW. Our study results rely on the precision of measured urine CrCL and it is not possible to rule out the potential for incomplete urine collection. We did not record data on clinical and microbiological response to vancomycin. Only half of our studied patients had a documented infection with Gram-positive bacteria; therefore, determining whether optimizing antibiotic concentrations with CIV leads to improved outcomes requires further study. Last, we did not assess the adequacy of our loading dose and the effects on vancomycin concentrations at 24 hours.

## Conclusion

Utilization of CIV in obese patients consistently achieves target therapeutic serum concentrations and reduces the total weight-based daily exposure of vancomycin compared to non-obese patients. CIV clearance is similar in obese and non-obese patients and does not increase proportionally to TBW.

## Abbreviations

ABW, adjusted body weight; APACHE II, acute physiology and chronic health evaluation; AUC24, area under the concentration curve 24 h; BMI, body mass index; CG CrCL, Cockcroft-Gault creatinine clearance; CI, continuous infusion; CIV, vancomycin continuous infusions; CLvanc, CI vancomycin clearance; CrCL, creatinine clearance; CVVH, continuous venovenous hemofiltration; IBW, ideal body weight; MRSA, methicillin-resistant *Staphylococcus aureus*; MSSA, methicillin-sensitive *Staphylococcus aureus*; SCr, serum creatinine; SSTI, skin soft tissue infection; TBW, total body weight; Vd, volume of distribution
